# Microsatellite instability in prostate cancer by PCR or next-generation sequencing

**DOI:** 10.1186/s40425-018-0341-y

**Published:** 2018-04-17

**Authors:** Jennifer A. Hempelmann, Christina M. Lockwood, Eric Q. Konnick, Michael T. Schweizer, Emmanuel S. Antonarakis, Tamara L. Lotan, Bruce Montgomery, Peter S. Nelson, Nola Klemfuss, Stephen J. Salipante, Colin C. Pritchard

**Affiliations:** 1 Department of Laboratory Medicine, University of Washington, Seattle, WA USA; 2 Department of Medicine, Division of Medical Oncology, University of Washington, Seattle, WA USA; 3 Department of Oncology, Johns Hopkins University School of Medicine, Baltimore, MD USA; 4 Department of Urology, Johns Hopkins University School of Medicine, Baltimore, MD USA; 5 Department of Pathology, Johns Hopkins University School of Medicine, Baltimore, MD USA; 6 VA Puget Sound Health Care System, Seattle, WA USA; 7 Human Biology Division, Fred Hutchinson Cancer Research Center, Seattle, WA USA

**Keywords:** Prostate adenocarcinoma, Microsatellite instability, MSI, Promega, Capillary electrophoresis, Mismatch repair, NGS, Next-generation sequencing, mSINGS

## Abstract

**Background:**

Microsatellite instability (MSI) is now being used as a sole biomarker to guide immunotherapy treatment for men with advanced prostate cancer. Yet current molecular diagnostic tests for MSI have not been evaluated for use in prostate cancer.

**Methods:**

We evaluated two next-generation sequencing (NGS) MSI-detection methods, MSIplus (18 markers) and MSI by Large Panel NGS (> 60 markers), and compared the performance of each NGS method to the most widely used 5-marker MSI-PCR detection system. All methods were evaluated by comparison to targeted whole gene sequencing of DNA mismatch-repair genes, and immunohistochemistry for mismatch repair genes, where available.

**Results:**

In a set of 91 prostate tumors with known mismatch repair status (29-deficient and 62-intact mismatch-repair) MSIplus had a sensitivity of 96.6% (28/29) and a specificity of 100% (62/62), MSI by Large Panel NGS had a sensitivity of 93.1% (27/29) and a specificity of 98.4% (61/62), and MSI-PCR had a sensitivity of 72.4% (21/29) and a specificity of 100% (62/62).

**Conclusions:**

We found that the widely used 5-marker MSI-PCR panel has inferior sensitivity when applied to prostate cancer and that NGS testing with an expanded panel of markers performs well. In addition, NGS methods offer advantages over MSI-PCR, including no requirement for matched non-tumor tissue and an automated analysis pipeline with quantitative interpretation of MSI-status.

**Electronic supplementary material:**

The online version of this article (10.1186/s40425-018-0341-y) contains supplementary material, which is available to authorized users.

## Background

Microsatellite instability (MSI) is characterized by mutations in repetitive DNA sequence tracts, caused by a failure of the DNA mismatch repair system to correct these errors. Deficient DNA mismatch repair (dMMR) results from the bi-allelic mutational inactivation or epigenetic silencing of any of the genes in the MMR pathway (most commonly *MSH2, MSH6, MLH1*, and *PMS2*). Consequently, MSI status is used as a biomarker indicative of dMMR. In May of 2017, the U.S. Food and Drug Administration (FDA) granted accelerated approval of an immunotherapy-based anti-PD-1 cancer treatment (pembrolizumab) for patients whose cancers have MSI or dMMR [1]. This is the first time the FDA has approved a drug based on the genetic characteristics of a tumor alone, regardless of the tumor’s original location (“tumor agnostic”).

MSI has been most closely studied in colorectal cancers, where it is present in up to 15–20% of cases [2]. However MSI has been found in many cancer types including endometrial (26–33%), ovarian (10%), cervical (8%), and gastric (8–22%) [3–9]. In prostate cancer, MSI and dMMR have been reported in a subset of tumors ranging from ~ 1% in primary to up to 12% of metastatic cancers [10–13].

Prostate cancer is the third leading cause of cancer death in American men and survival rates are low for prostate cancers that advance to metastatic castration-resistant disease [14]. Despite recent advances and a range of treatment options for metastatic castration-resistant prostate cancer (mCRPC), outcomes are varied and clinicians are not able to predict response to the available therapies [15]. Predictive biomarkers, which can tailor the treatment of mCRPC to individual patients, are urgently needed. MSI a promising marker for evaluation in prostate cancer, with robust responses to pembrolizumab reported in MSI advanced mCRPC [16]. However, current MSI-detection assays have not been evaluated for use in prostate cancer. The original National Cancer Institute (NCI) recommended 5-marker microsatellite panel, commonly known as the “Bethesda panel,” was developed in 2004 to screen patients for hereditary non-polyposis colorectal cancer (also known as Lynch syndrome) [17, 18]. The original NCI report makes clear that “the [Bethesda] reference panel is recommended for the characterization of MSI in colorectal cancer only” [17]. This panel is the basis for the most widely used MSI detection assay, the Promega MSI Analysis System [18]. While subsequent studies have shown the efficacy of using the Bethesda panel to test for MSI in other cancer types, specifically endometrial cancers [19], other studies have reported that the instability of microsatellite loci can vary greatly across different cancer types [3].

Yet regardless of tumor type, conventional MSI testing is still routinely performed using PCR and fragment analysis of the 5-marker Bethesda panel (BAT-25, BAT-26, MONO-27, NR-21 and NR-24) [17, 18] and/or through immunohistochemical (IHC) detection of MMR proteins. IHC also has diagnostic limitations [20]. For example, IHC cannot always detect loss of mutated proteins resulting from missense mutations and can have normal staining even for some protein-truncating mutations [21]. Consequently, there is an immediate need for a highly sensitive and specific diagnostic assay for MSI targeted to specific tumor types.

To address this need in prostate cancer, we evaluated two next-generation sequencing (NGS) MSI-detection methods, MSIplus [22] and MSI by Large Panel NGS [23], for efficacy in prostate cancer and compared the performance characteristics of each method to the MSI-PCR based on the 5-marker Bethesda panel [18] (Promega, Madison, WI, USA). Concurrently, we compared both NGS methods and MSI-PCR to targeted deep sequencing of MMR genes: because dMMR is caused by bi-allelic gene mutation rather than epigenetic silencing in prostate cancer [11], deep sequencing can provide a definitive “gold-standard” diagnosis.

## Methods

### Patients and specimens

A total of 91 prostate tumor samples were analyzed from 4 different sources: 1) Primary and metastatic prostate cancer tissue from the University of Washington (UW) Prostate Cancer Donor Rapid Autopsy Program (*n* = 31) [11], 2) LuCaP patient-derived tumor xenografts (PDX) (*n* = 23) [11, 24], 3) UW-OncoPlex prostate cancer precision medicine program (*n* = 28) [25], and 4) Johns Hopkins University (*n* = 9) [26]. All tumors had > 20% neoplastic cellularity. Of the 91 total samples, 80 represent unique patients or xenografts. The 11 non-unique specimens represent primary and metastatic tissues from the same patient, LuCaP PDX subtypes (castration-resistant or not), or LuCaP PDX derived from patient samples. Genomic DNA was prepared from either formalin-fixed paraffin-embedded tissue (FFPE, *n* = 71) or fresh-frozen tissue (*n* = 20) with the Gentra Puregene DNA Isolation Kit (Qiagen, Catalog #158489). Clinical specimens were obtained in accordance with the declaration of Helsinki and the ethics guidelines of the human subjects division of the University of Washington and Johns Hopkins University.

### Microsatellite instability by MSIplus

The MSIplus panel includes the 5 microsatellite markers that comprise the Promega MSI Analysis System plus an additional 13 discriminatory microsatellite markers which are frequently unstable in MSI positive tumors (Table 1). Detection of MSI status by MSIplus was performed by the CLIA-certified UW clinical genetics and solid tumors laboratory as described in Hempelmann et al. [22] following a standard operating procedure that was clinically validated to assess MSI status in colorectal samples (see Additional file 1 for detail). Based on established guidelines for small panels of selected discriminatory microsatellite markers, a fraction of ≥ 0.33 (≥ 33% unstable loci) was considered MSI-positive [17, 27–30].

**Table 1:** MSIplus and MSI-PCR Microsatellite Loci

Target Loci	Panel(s)	Loci Coordinates (GRCh37/hg19)	Repeat Type	Gene
Bat25	MSI-PCR, MSIplus	chr4:55598212–55598236	(T)25	KIT, intronic
Bat-26	MSI-PCR, MSIplus	chr2:47641560–47641586	(A)27	MSH2, intronic
MONO-27	MSI-PCR, MSIplus	chr2:39564894–39564921	(T)28	MAP4K3, intronic
NR-21	MSI-PCR, MSIplus	chr14:23652347–23652367	(A)21	SLC7A8, exonic
NR-24	MSI-PCR, MSIplus	chr2:95849362–95849384	(T)23	ZNF2, exonic
MSI-01	MSIplus	chr1:201754411–201754427	(T)17	NAV1, intronic
MSI-03	MSIplus	chr2:62063094–62063110	(A)17	FAM161A, intronic
MSI-04	MSIplus	chr2:108479623–108479640	(T)18	RGPD4, intronic
MSI-06	MSIplus	chr5:172421761–172421775	(T)15	ATP6V0E1, intronic
MSI-07	MSIplus	chr6:142691951–142691967	(T)17	GPR126, intronic
MSI-08	MSIplus	chr7:1787520–1787536	(A)17	ELFN1, exonic
MSI-09	MSIplus	chr7:74608741–74608753	(T)13	GTF2IP1, intronic
MSI-11	MSIplus	chr11:106695515–106695526	(T)12	GUCY1A2, intronic
MSI-12	MSIplus	chr15:45897772–45897785	(T)14	BLOC1S6, intronic
MSI-13	MSIplus	chr16:18882660–18882674	(A)15	SMG1, intronic
MSI-14	MSIplus	chr17:19314918–19314935	(T)18	RNF112, intronic
HSPH1-T17	MSIplus	chr13:31722621–31722637	(A)17	HSPH1, intronic
EWSR1	MSIplus	chr22:29696469–29696484	(T)16	EWSR1, exonic

For coordinates of the loci captured by Large-Panel NGS see Additional file 2: Table S1

### Microsatellite instability by large panel NGS

MSI was assessed from BROCA or UW-OncoPlex sequence data [11, 31] using mSINGS as previously described by Salipante et al. [23]. This method evaluates microsatellite loci that are incidentally captured during targeted sequencing of gene panels (146 mononucleotide microsatellite loci captured by BROCA and 65 mononucleotide microsatellite loci captured by UW-OncoPlex) (Additional file 2: Table S1). Based on previous validation studies, a fraction of > 0.20 (> 20% unstable loci) was considered MSI-positive [23]. This threshold is lower than that used for MSIplus (> 0.33) because Large Panel NGS evaluates unselected microsatellites, whereas MSIplus evaluates loci selected for their ability to distinguish MSI-negative and MSI-positive samples, conferring high discriminatory power.

### Microsatellite instability by MSI-PCR

Testing was performed by the UW clinical genetics and solid tumors laboratory using the 5-marker MSI Analysis System v1.2 (Promega, Madison, WI, USA) following a clinically-validated standard operating procedure based on the manufacturers’ protocol (see Additional file 1 for detail). All MSI-PCR electropherograms were analyzed by at least 2 independent reviewers for evidence of MSI. Specimens demonstrating instability within 2 or more of the 5 markers were considered ‘MSI-positive’ and all others were considered ‘MSI-negative’. We did not classify any specimens as ‘MSI-low’ given the accumulation of evidence showing that it is not reliable when examining a small number of loci [3, 27, 32].

### Limit of detection of MSI assays

Previous clinical validation of MSIplus, Large Panel NGS, and MSI-PCR completed by the UW clinical genetics and solid tumors laboratory established the limit of detection at 20% tumor-cellularity for all assays.

### Targeted sequencing by large panel NGS

Targeted deep sequencing of DNA mismatch repair genes was performed by the UW clinical genetics and solid tumors laboratory using either the BROCA or UW-OncoPlex Large Panel NGS assays as previously described [11, 31].

### Immunohistochemistry

A subset of 21 autopsy samples were tested for expression of MMR proteins by immunohistochemistry (IHC) tissue microarray as described by Pritchard et al. [11] and Nghiem et al. [33].

## Results

### Establishing a validation sample set

We selected validation samples from 4 prostate cancer series compromising primary and metastatic disease. dMMR is caused by bi-allelic gene mutation rather than epigenetic silencing in prostate cancer [11]. Therefore, we established “gold standard” dMMR status based on targeted deep gene sequencing (*n* = 91 total; *n* = 29 with bi-allelic dMMR inactivating mutations, and *n* = 62 without) (Additional file 2: Table S2). For all further analyses, samples with bi-allelic dMMR mutations were considered true MSI-positives and samples without bi-allelic dMMR mutations were considered true MSI-negatives.

### Microsatellite instability by MSIplus

**Fig. 1:**
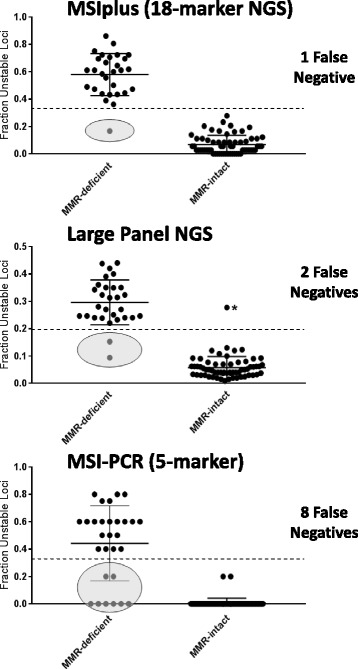
Performance Comparison of MSI methods in Prostate Cancer. Specimens are categorized as either MMR-deficient (*n* = 29) or MMR-intact (*n* = 62) based on targeted deep sequencing of DNA mismatch repair genes. Ovals specify false negative results. Error bars show mean and standard deviation. The dashed lines indicate the threshold delineating MSI-positive from MSI-negative. Specimens above the lines are interpreted as MSI-positive and specimens below the lines are interpreted as MSI-negative. NOTE: Large panel NGS has a lower threshold for positivity (> 0.20) than small panel MSI assays (MSI-PCR and MSIplus) that measure only selected highly-discriminatory loci. These thresholds have been validated as described in the methods. Asterisk indicates single false positive by Large Panel NGS (sample C03)

MSIplus had a sensitivity of 96.6% (28 of 29 MSI-positive cases identified; 95% CI, 80.4%–99.8%), and a specificity of 100% (62 of 62 MSI-negative cases identified; 95% CI, 92.7%-100) (Table 2) (Fig. 1a) (Additional file 2: Table S2).

**Table 2:** Performance Characteristics of MSIplus, Large-Panel NGS, and MSI-PCR in Prostate Cancer

Assay	Sensitivity [95% CI]	Specificity [95% CI]
MSIplus	96.6% [80.4%–99.8%]	100% [92.7%–100%]
Large-Panel NGS	93.1% [75.8%–98.8%]	98.4% [90.2%–99.9%]
MSI-PCR	72.4% [52.5%–86.6%]	100% [92.7%–100%]

All samples typed by MSIplus were run in duplicate, yielding a set of 182 separately prepared and sequenced within-run replicates with which to evaluate reproducibility (Additional file 2: Table S3). Thirty-five percent of the replicates had identical mSINGS scores. The average absolute difference between technical replicates was 0.06 mSINGS units. There were two instances of discordant MSIplus interpretations between technical replicates (sample A10, mSINGS scores: 0.3529 and 0.1111; and sample L12, mSINGS scores: 0.3333 and 0.2222). After averaging the mSINGS scores of the replicates (see Additional file 1), both samples were correctly classified as MSI-negative.

### Microsatellite instability by large panel NGS

Examining the same 91 prostate specimens, we found that Large Panel NGS had a sensitivity of 93.1% (27 of 29 MSI-positive cases identified; 95% CI, 75.8%–98.8%) and a specificity of 98.4% (61 of 62 MSI-negative cases identified; 95% CI, 90.2%–99.9%) (Table 2) (Fig. 1b) (Additional file 2: Table S2).

### Microsatellite instability by MSI-PCR

For the same set of samples, MSI-PCR had a sensitivity of 72.4% (21 of 29 MSI-positive cases identified; 95% CI, 52.5%–86.6%) and a specificity of 100% (62 of 62 MSI-negative cases identified; 95% CI, 92.7%–100%) (Table 2) (Fig. 1c) (Additional file 2: Table S2). While a consensus was reached for all 91 specimens, initial interpretations produced discordant results for two MSI-negative samples (A10 and A14). Director-A interpreted both samples as MSI-negative and director-B interpreted both as MSI-positive (Additional file 2: Table S4). After three independent laboratory directors (A, B, & C) reviewed the discordant samples as a group, all directors interpreted the samples as MSI-negative. There were an additional 10 samples for which the directors’ overall diagnosis was concordant (either negative or positive) but the total number of unstable loci was discordant (Additional file 2: Table S4).

### Correlation with immunohistochemistry (IHC)

Although our primary objective was to compare the performance of expanded-marker NGS assays to MSI-PCR, we also evaluated the performance of IHC when this information was available (Additional file 2: Table S2). For the 21 samples with corresponding IHC data, gene sequencing confirmed that 7 were MMR-deficient and 14 MMR-intact. The results of MSI-testing by MSIplus, Large Panel NGS, and MSI-PCR were concordant for these 21 samples (7/7 MMR-deficient were MSI-positive; 14/14 MMR-intact were MSI-negative). The IHC results were consistent with the findings of the other approaches in all but 2 of the 21 cases. One MMR-intact, MSI-negative autopsy sample (A03) had isolated loss of MSH6 by IHC, and another MMR-intact, MSI-negative autopsy sample (A18) had isolated loss of PMS2 by IHC [11, 33].

### Concordance between tissue sites

We evaluated concordance between tissue sites in one pair of autopsy samples (A24.1 & A24.2) from different metastatic sites, a second pair of autopsy samples (A28.1 & A28.2) from primary and metastatic tumor sites, and in 8 pairs of LuCaP PDX samples which included both the original xenograft lines and castration-resistant (CR) lines. In these samples MSI status was always concordant between metastatic sites, between primary and metastatic tumor, and between CR and non-CR PDX lines for all MSI detection methods (Additional file 2: Table S2).

## Discussion

We found that the conventional MSI-PCR method developed and validated for colon cancer has inferior sensitivity when applied to prostate cancer, and that NGS testing with an expanded panel of markers performs more robustly. For our test set, the 5-marker Bethesda Panel (MSI-PCR) had a sensitivity of only 72.4% whereas the expanded 18-marker NGS assay MSIplus had a sensitivity of 96.6% and Large Panel NGS had a sensitivity of 93.1%. Specificity was high for all approaches: neither MSI-PCR nor MSIplus generated any false positive results, and Large Panel NGS generated only one false positive (Fig. 1). Predictive modeling estimates that by the year 2020, there will be over 40,000 men in the United States with mCRPC and that more than half of those patients will see their disease progress after exhausting standard treatment options [34]. A systematic review of available epidemiological data supports these estimates [14]. Even if MSI is present in only ~ 5% of these patients, using an MSI approach that is more sensitive could qualify many more men for life-extending immunotherapy.

In addition to improved sensitivity, NGS-based MSI testing with MSIplus or Large-panel NGS offers several advantages over conventional capillary electrophoresis MSI-PCR methods. 1) *they do not require matched non-tumor tissue.* MSI-PCR typically requires a matched-normal DNA sample for every specimen assayed because rare germline polymorphisms may be misinterpreted as positive microsatellite loci. Likewise, some NGS-based MSI-detection methods such as MANTIS [35] and MSIsensor [36] require a matched-normal sample. Neither MSIplus nor Large Panel NGS require matched-normal DNA, which helps to simplify testing logistics and reduce cost. *2) Interpretation is streamlined and semi-automated.* The interpretation of MSIplus and Large Panel NGS is completed by an automated analysis pipeline based on quantitative statistics (mSINGS) [23], whereas interpretation of MSI-PCR data is both manual and qualitative. Automation greatly reduces analysis time and is likely to reduce inter-observer and inter-laboratory variation. Conversely, the inter-laboratory variability of MSI-PCR has been systemically demonstrated by the College of American Pathologists (CAP): results of the 2011 CAP microsatellite instability 5-marker capillary electrophoresis data interpretation survey which tested 88 independent laboratories revealed that only 78% of laboratories correctly identified an MSI-positive specimen [37].

Our study has several limitations that should be addressed in future work. It was not designed to assess the diagnostic accuracy of mismatch repair protein IHC in prostate cancer, which is a mainstay of testing for purpose of inferring MSI status in many pathology laboratories. In addition, we do not have information on responses to pembrolizumab or other clinical outcomes in our validation set. Our study design does not permit estimation of positive or negative predictive value because the samples are not part of a consecutive prospective series. Finally, we did not assess total mutational burden, which is another emerging biomarker which has been associated with mutations in MMR genes and sensitivity to immune checkpoint inhibitors [38–40].

## Conclusions

In summary, our study demonstrates that expanded panel MSI NGS assays performed well in prostate cancer, and that the conventional 5-marker capillary electrophoresis MSI-PCR assay had inferior sensitivity. Both NGS assays we studied were superior to MSI-PCR, however MSIplus is probably most appropriate as first-line MSI screening in a low-prevalence population. MSIplus is an amplicon-based NGS assay, which takes less time, uses less sample material, and is much lower cost than Large Panel NGS. Given the ever-increasing demand for clinical MSI testing following the FDA’s tumor agnostic approval of pembrolizumab for MSI-positive cancers, a low-cost, relatively fast, and highly sensitive/specific assay for MSI is urgently needed. MSIplus fulfills all these criteria and is now validated for both colorectal and prostate cancer. The MSIplus assay could be further improved by adding microsatellite loci that are especially informative in prostate cancer.

## Additional files

Additional file 1: Supplementary Methods. (DOCX 20 kb)

Additional file 2: **Table S1.** Large-Panel NGS Microsatellite Loci. **Table S2.** Detail on Samples, Targeted Sequencing, Microsatellite Instability, and IHC (when available). **Table S3.** Detail on MSIplus Technical Replicates. **Table S4.** Discordant Interpretation of MSI-PCR (Promega 5-marker). **Table S5-A.** MSIplus Stage-1 PCR Primers. **Table S5-B.** MSIplus Stage-2 PCR Primers. **Table S5-C.** MSIplus Custom Index Sequencing Primer. (XLSX 143 kb)

## Abbreviations

CAP: College of American Pathologists
CI: Confidence-interval
CLIA: Clinical laboratory improvement amendments
CR: Castration-resistant
dMMR: Deficient DNA mismatch repair
FDA: US Food and Drug Administration
FFPE: Formalin-fixed paraffin-embedded tissue
IHC: Immunohistochemistry
mCRPC: Metastatic castration-resistant prostate cancer
MMR: DNA Mismatch repair
MSI: Microsatellite instability
NCI: National Cancer Institute
NGS: Next-generation sequencing
PDX: Patient-derived tumor xenograft
UW: University of Washington
