# Supplementary material for: Microsatellite instability in prostate cancer by PCR or next-generation sequencing
Source: J Immunother Cancer. 2018 Apr 17;6:29. doi: 10.1186/s40425-018-0341-y (PMC5904988; doi:10.1186/s40425-018-0341-y)
Supplement: Supplementary file 1 — Supplementary Methods. (DOCX 20 kb) [file 40425_2018_341_MOESM1_ESM.docx]

**Supplementary Methods**

**Microsatellite Instability by MSIplus.** Sequencing libraries were generated by PCR amplification in two separate stages. The purpose of the first stage was to simultaneously amplify the microsatellite loci of interest and to incorporate partial Illumina sequencing adaptors into the amplified product. The second stage of PCR fully extended the sequencing adaptors and incorporated unique 8-bp, sample-specific index sequences, which enabled the multiplexing of multiple specimens together onto the same sequencing run. As described in Hempelmann et al. [1], the MSIplus assay can be used to evaluate mutations in relevant cancer genes (*KRAS*, *NRAS*, and *BRAF*) by simultaneously amplifying the trio of cancer genes and the 18 microsatellite loci. However, in the present study, the genes were not analyzed and we focused exclusively on the microsatellite loci. All samples were processed in duplicate and each run included a negative, positive, and no-template control.

The first stage of PCR was performed in a 50-μL volume reaction using the Qiagen Multiplex PCR Kit (Qiagen, Valencia, CA), incorporating 50-ng template DNA, 5-uL of the 18-plex microsatellite primer pool, 1x Qiagen PCR Master Mix, and 1× Qiagen Q-solution. The final concentration of each microsatellite primer in the primer pool was: 1.5 μmol/L for the EWSR1 primer pair (EWSR1-fwd and EWSR1-rev) and 0.75 μmol/L for the remaining 17 primer pairs (Supplementary Table S5). PCR cycling conditions were as follows: 15 minutes incubation at 95°C; 30 cycles of 94°C for 30 seconds, 60°C for 90 seconds, and 72°C for 60 seconds; and a final extension at 72°C for 10 minutes. Before the second stage of PCR, amplification products were purified using a 0.8× volume of Agencourt AMPure XP magnetic beads (Beckman-Coulter, Indianapolis, IN), according to the manufacturer's instructions.

The second stage of PCR was performed using 5 ng of amplification product from the first stage of PCR as template. PCR was performed in a 50-μL volume reaction using KAPA HiFi HotStart ReadyMix PCR Kit (KAPABiosystems, Wilmington, MA) and 0.3 μmol/L of each of the second-stage primers (Supplementary Table S6). PCR cycling conditions were as follows: 3 minutes incubation at 95°C; five cycles of 98°C for 20 seconds, 65°C for 15 seconds, and 72°C for 60 seconds; and a final extension at 72°C for 5 minutes. PCR products were purified using a 1.8× volume of Agencourt AMPure XP magnetic beads.

Sequencing was performed on an Illumina MiSeq (San Diego, CA) using 200-bp, single-ended reads and an 8-bp index read, with the addition of a custom index sequencing primer (Supplementary Table S6). Sequence reads were processed using an automated data analysis pipeline as described in Hempelmann et al. 2015 [1]. MSI status was determined using the mSINGS analysis package [2] (source code at: https://bitbucket.org/uwlabmed/msings). For each specimen, microsatellite loci covered by a read depth of <30 × were excluded as not passing quality filter. Post pipeline and mSINGS analysis, sample replicates were compared. The fraction of unstable loci (the mSINGS score) was averaged prior to MSI status determination if the quality of both replicates was high (> 9 loci passed quality filter). Replicates with less than 9 loci passing quality filter were excluded from further analysis.

**Microsatellite Instability by Promega MSI Analysis System (MSI-PCR)**. Approximately 50ng of genomic DNA was amplified in a 10uL total reaction containing 0.075 units Taq Polymerase (New England BioLabs, Beverly, MA), 1x TaqStart Antibody (Clontech-Takara Bio, Mountain View, CA ), 1x Promega MSI Gold Buffer and 1x Promega MSI Primer Mix (Promega, Madison, WI, USA). PCR cycling conditions followed the manufacturers recommendations and were as follows: 11 minutes incubation at 95°C followed by 60 seconds incubation at 96°C; 10 cycles of 94°C for 30 seconds, 58°C for 30 seconds, and 70°C for 60 seconds; 25 cycles of 90°C for 30 seconds, 58°C for 30 seconds, and 70°C for 60 seconds; followed by a final extension step of 60°C for 30 minutes. For each sample, amplified products were loaded neat and diluted 1:20 for fragment analysis by capillary electrophoresis using an ABI PRISM® 3100 Genetic Analyzer and GeneScan 500 ROX size standard (Applied Biosystems, Carlsbad, CA). Each PCR and electrophoresis included a known negative control, positive control, and no-template-control.

**REFERENCES**

1. Hempelmann JA, Scroggins SM, Pritchard CC, Salipante SJ. MSIplus for Integrated Colorectal Cancer Molecular Testing by Next-Generation Sequencing. J. Mol. Diagn. JMD. 2015;17:705–14.

2. Salipante SJ, Scroggins SM, Hampel HL, Turner EH, Pritchard CC. Microsatellite instability detection by next generation sequencing. Clin. Chem. 2014;60:1192–9.
